# Methodology for development of an expert system to derive knowledge from existing nature-based solutions experiences^✰^

**DOI:** 10.1016/j.mex.2022.101978

**Published:** 2022-12-21

**Authors:** Shahryar Sarabi, Qi Han, Bauke de Vries, A.Georges L. Romme

**Affiliations:** a Information Systems in the Built Environment (ISBE) Group, Department of Built Environment, Eindhoven University of Technology, Groene Loper 3, 5612 AE Eindhoven, Netherlands; b Department of Industrial Engineering & Innovation Sciences, Eindhoven University of Technology, Groene Loper 3, 5612 AE Eindhoven, Netherlands

**Keywords:** Nature-based solutions (NBS), Expert system, Knowledge acquisition, Artificial intelligence, case-based reasoning, Hybrid expert system integrating a black-box Artificial Neural Network(ANN) with a white-box Case-Based Reasoning (CBR) model

## Abstract

Learning from past experiences is essential for the adoption of Nature-Based Solutions (NBS). There is a growing number of knowledge repositories sharing the experience of NBS projects implemented worldwide. These repositories provide access to a large amount of information, however, acquiring knowledge from them remains a challenge. This paper outlines the technical details of the NBS Case-Based System (NBS-CBS), an expert system that facilitates knowledge acquisition from an NBS case repository. The NBS-CBS is a hybrid system integrating a black-box Artificial Neural Network (ANN) with a white-box Case-Based Reasoning model. The system involves:

• a repository that stores the information of past NBS projects, and an input collection component, guiding the collection and encoding of the user's inputs;
• a classifier that predicts solutions (i.e., generates a hypothesis), based on user input (target case), drawing on a pre-trained ANN model to guide the case retrieval, and a case retrieval engine that identifies cases similar to the target case;
• a case adaption and retainment process in which the user assesses the provided recommendations and retains the solved problem as a new case in the repository.

## Graphical abstract

**Figure:**
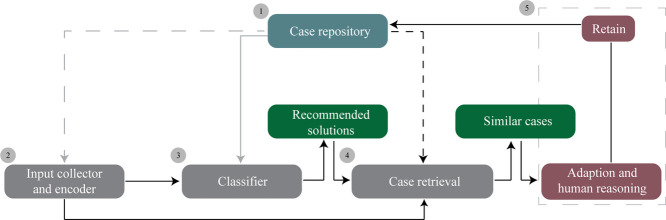

Specifications table

**Table:** 

Subject area:	
More specific subject area:	Environmental information systems
Name of your method:	Hybrid expert system integrating a black-box Artificial Neural Network(ANN) with a white-box Case-Based Reasoning (CBR) model
Name and reference of original method:	There is no specific method that was originally developed and then modified. We applied different methods used in other domains to develop an information system for facilitating knowledge extraction from an NBS experience repository. For developing the ANN model, we used the TensorFlow 2.7 python library (Abadi et al., 2016), and for text mining and developing the CBR engine, we used the scikit-learn python library (Pedregosa et al., 2011).
Resource availability:	The case repository used in the expert system presented here is not currently publicly available. The updated NBS-CBS (Urban Nature Recommender) URL can be found here: (https://github.com/Shahryar73/Urban-Nature-Recommender).

## Background

The uptake and planning of Nature-Based Solutions (NBS), as complex integrative solutions, requires knowledge from multiple disciplines [10]. Information systems for NBS enable users to access information and facilitate knowledge acquisition. There have been several efforts to develop information systems that support the NBS planning process (see [12]). However, most of these systems deal with a common problem: they usually rely on “if-then” rules and models to derive recommendations, while NBS planning involves major uncertainties and collecting the required information is difficult, especially in urban settings. In such conditions, learning from experience can have many advantages [4]. Multiple repositories provide information regarding past NBS experiences (e.g. OPPLA platform, NetworkNature resource platform, Nature4Cities, Nature-based Solutions Policy Platform, Nature-based Solutions Evidence Platform, Climate-ADAPT). These repositories provide a large amount of information about different aspects of the implementation process in each NBS case. However, finding the relevant information and gaining knowledge from these repositories remains a challenge.

Acquiring knowledge from such repositories can be facilitated using machine learning models. These models can be classified into two general categories: (1) black-box models that are capable of generating results with relatively high accuracy but are difficult to understand [11], and (2) white-box models that are easy to understand but may not be as efficient and accurate in providing results [7]. This article describes the technical details of the NBS Case-Based System (NBS-CBS), which integrates a black-box Artificial Neural Network Model with a White-box Case-Based Reasoning model to exploit the advantages of both types of machine learning models.

## Method details

**Fig. 1:**
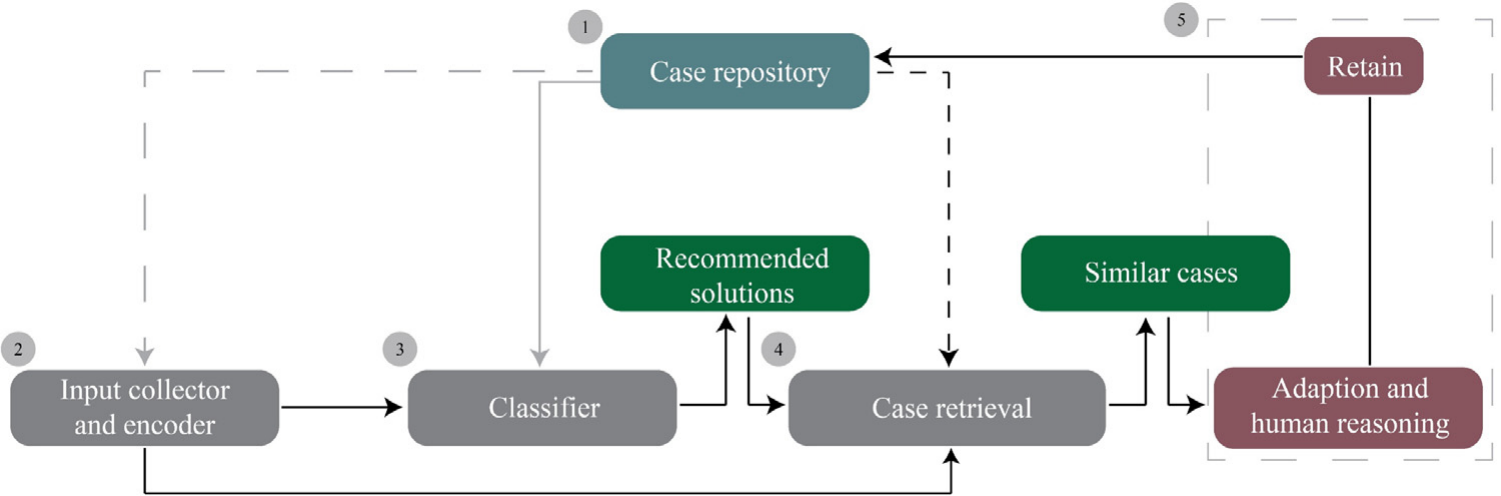
NBS-CBS framework. Source: [13]. Note: solid black arrows show the main processes that users go through; the solid gray arrow shows the classifier is trained on the case repository, while there is no dynamic interaction between the two; the dashed gray arrow shows the case repository's structure dictates the encoding function (again with no dynamic interaction); the dashed black arrow shows the case retrieval component receives data from the case repository.

The NBS-CBS is a hybrid system integrating a black-box Artificial Neural Network (ANN) with a white-box Case-Based Reasoning model. This system, relying on information from past NBS experiences, provides users with recommendations regarding suitable NBS measures to consider and finds similar past projects that provide users with relevant information regarding the opportunities and challenges of uptaking NBS. This system involves five components, as also shown in Fig. 1:

1. A repository that stores the information of past NBS projects;
2. An input collection component, guiding the collection and encoding of the user's inputs;
3. A classifier that predicts solutions (i.e., generates a hypothesis), based on user input drawing on a pre-trained ANN model to guide the case retrieval in the next step;
4. A case retrieval engine that retrieves cases similar to the target case, based on user input and the solutions recommended by the ANN model;
5. A case adaption and retain process in which the user judges the relevance of the provided recommendations, assesses the adaptability of these recommendations to the target context, and retains the solved problem as a new case in the repository.

### Case repository

**Fig. 2:**
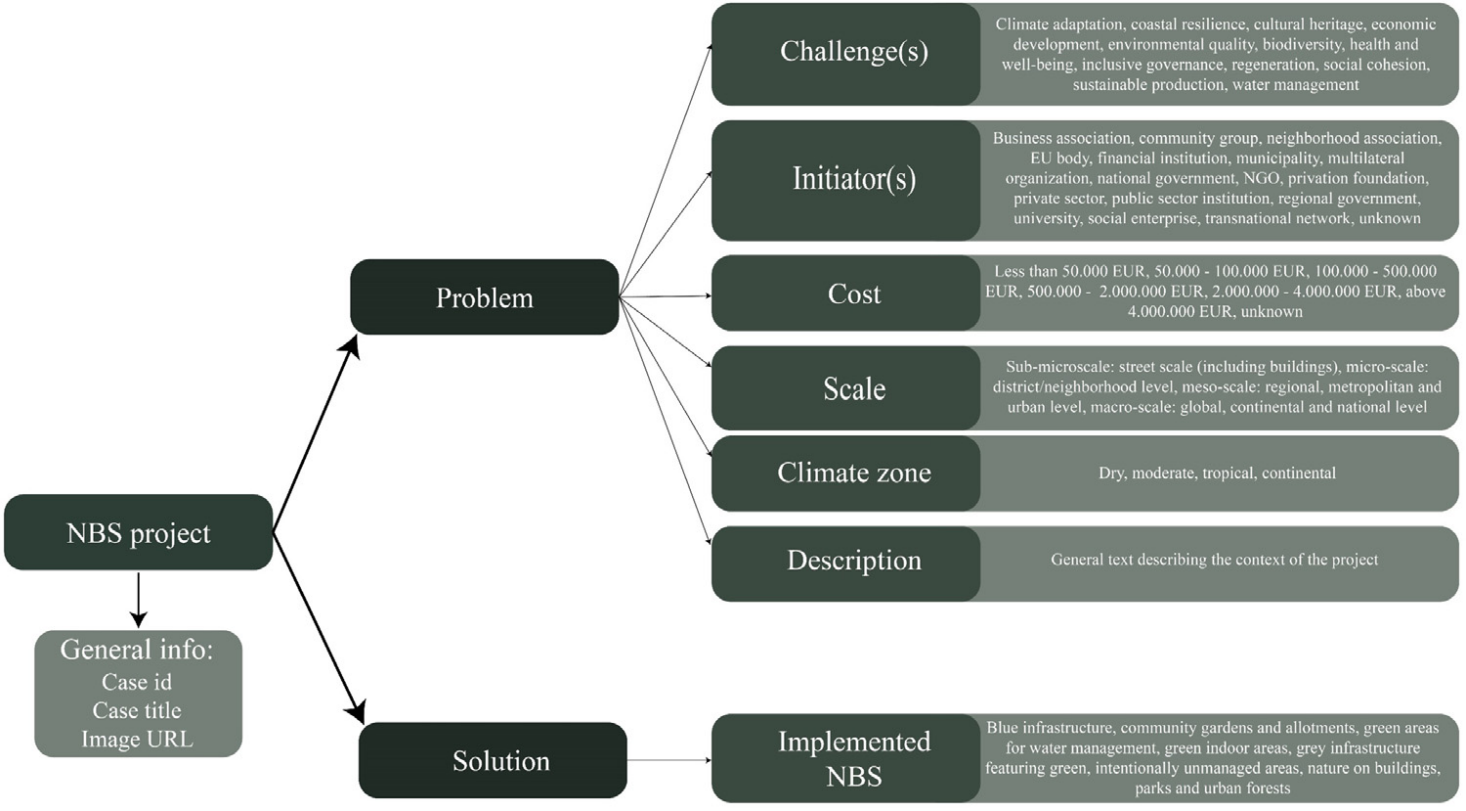
Case data structure.

The first task in developing a CBS is to create a case repository. The case repository is populated with the information from the Urban Nature Atlas (UNA) database. UNA is one of the largest NBS project databases containing detailed structured and unstructured information about more than 1100 implemented NBS projects [2], which makes it suitable to serve as the knowledge base of the NBS-CBS. Cases are represented by two types of features (see Fig. 2): problem and solution features. The problem features describe the context and the conditions of each case and include: the sustainability challenge(s) aimed to be addressed by the projects, project initiator(s), cost of the project, scale of the project, climate zone where the case is located, and a general project description. The solution feature describes the entire solution adopted, including the featured NBS measures. The term ‘solution’ here does not refer to NBS, but to a solution for a defined problem that the system seeks to identify. Besides the problem and solution features, general information for each case, including “case id”, “case title” and “case image URL” are stored in the repository.

**Fig. 3:**
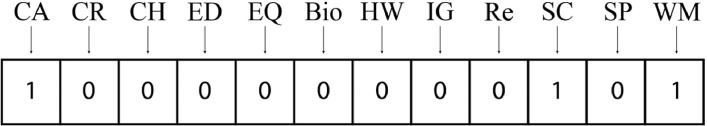
Multi-hot representation of challenge(s) feature. CA: Climate Adaptation, CR: Coastal Resilience, CH: Cultural Heritage, ED: Economic Development, EQ: Environmental Quality, Bio: Biodiversity, HW: Health and Well-being, IG: Inclusive Governance, *Re*: Regeneration, SC: Social Cohesion, SP: Sustainable Production, WM: Water Management.

The next step is to efficiently record and store features in the repository, in a format that can be processed by computer systems. Fig. 2 presents the features describing each case and the values considered for each feature. *Scale* and *cost* can be treated as ordinal variables and normalized (between 0 and 1) and represented by a single numerical value. The *climate zone* is a multi-class categorical feature that is represented using the one-hot encoding method [3]. Therefore, the climate zone feature involves a list of four binary values, each representing a climate zone (see Fig. 2). *Challenges, initiators*, and *implemented NBS* are multi-label categorical features, meaning each case can have multiple instances of a feature; therefore, the multi-hot encoding method was used [14] to represent these features. In Fig. 3, an example of multi-hot encoding of the challenge(s) feature is presented. This feature is represented by a list of 12 binary values (see), each indicating a potential challenge. There can be multiple elements with a value of 1 in this list, meaning multiple challenges have been addressed.

As a text feature, the *project* description needs to be represented numerically to be used in the analysis, a process commonly known as text vectorization. A widely used text vectorization method is the Term Frequency-Inverse Document Frequency (TF-IDF) which reflects the importance of a word in a document in a collection of documents [8]. This method assigns a value to each word in a document based on the word frequency while offsetting the value based on the frequency of the word among all documents (see Appendix A). After preprocessing the text (see Appendix A), a TF-IDF vectorizer was trained using the scikit-learn python library [9]. Subsequently, a matrix of TF-IDF values with n (i.e., the number of cases in the repository) rows was generated, each representing the project description of a case from the repository, and 6000 columns, each representing a unique term. Therefore, the problem description feature for each case involves an array of 6000 TF-IDF values. The trained vectorizer was stored to be reused in the next step. Table 1 shows the representation of the problem and solution features in the case repository.

**Table 1:** Representation of the problem and solution features in the case repository.

	Problem features						Solution feature
Case_id	Challenge(s) addressed (12 binary labels/ multi-hot encoding)	Initiator(s) (17 binary labels/ multi-hot encoding)	Scale (normalized numerical value)	Cost (normalized numerical value)	Climate Zone (4 binary labels/ one-hot encoding)	Project Description (6000 TF-IDF values (between 0 and 1))	Implemented NBS (8 binary labels)
1	[1,0,….,0,1]	[1,0,….,1]	0.66	0.4	[0,1,0,0]	[0.366,0,…,0.235,0]	[0,1,0,0,0,0,1,0]
2	[0,1,….,1,0]	[1,0,….,1]	0.33	0.2	[1,0,0,0]	[0,0,…,0.355,0]	[0,1,0,0,0,0,0,0]
…	…	…	…	…	…	…	…
n	[0,1,….,1]	[1,0,….,1]	1	0.8	[0,0,1,0]	[0,…, 0.564,0,…,0,0]	[0,1,0,0,1,0,0,1]

### Input collector and encoder

**Fig. 4:**
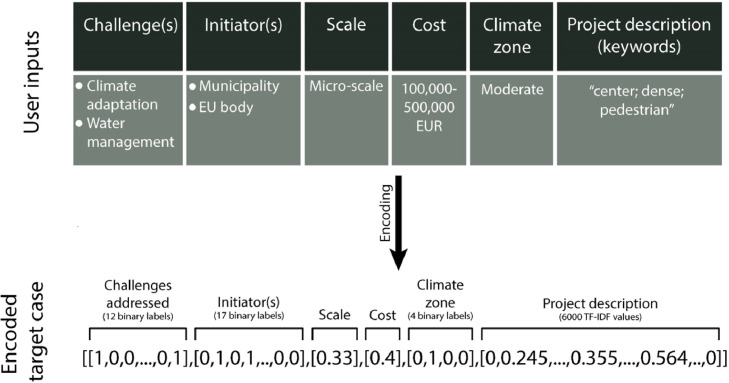
Example of encoding a target case.

For the system to be able to process and compare the target case with cases in the repository and for the classifier to provide a recommendation, the input data needs to be in the same format and structure as the cases stored in the case repository. At this step, users put in the information regarding the characteristics of their target case by providing the system with the information regarding the six problem features mentioned in the previous section. The encoding function will then transform the user's inputs into the same format and shape as the cases stored in the repository (see Fig. 4). Therefore, the multi-class categorical data will be encoded in the one-hot-encoded format, the multi-label categorical data in the format of multi-label binary, and text data (keywords) will be preprocessed and fed into the TF-IDF vectorizer trained in the previous step and transformed into an array of TF-IDF values with the same length as the text matrix of the case repository (6000 TF-IDF values).

### Classifier (ANN model)

This step aims to generate a hypothesis by providing an initial recommendation regarding the solution(s) to implement. The priority at this stage is to develop a classification model that can predict solutions with rather high accuracy. Moreover, given the amount and diversity of data involved, a black-box model that can adapt and identify the hidden patterns in the data is preferred at this stage. An ANN model was thus developed to perform this classification task and recommend NBS measures.

**Fig. 5:**
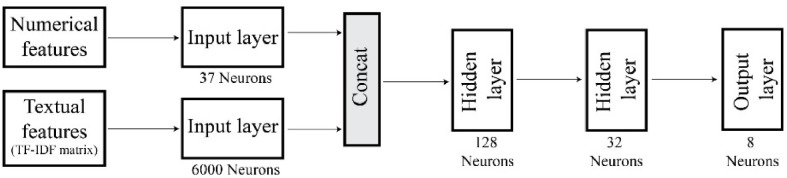
ANN architecture.

The ANN model was implemented as a feedforward neural network of four fully connected layers: an input layer (consisting of input neurons), two hidden layers (first one with 128 neurons and the second one with 32 neurons), and an output layer consisting of eight output neurons) (see Fig. 5). The model was developed using the TensorFlow 2.7 python library [1]. There are two types of input features fed into the model: tabular and textual features. The tabular features are all non-textual problem features joined into a single array. As described earlier, the textual feature (project description) for each case is represented by an array of TF-IDF values. These two input features (i.e., tabular and textual) are concatenated (referred to as Concat in Fig. 5) to generate the input layer (with 37 + 6000 neurons) for training the model. The output layer of the model consists of eight nodes representing the eight possible categories of NBS measures that can be predicted. The model provides the probability of occurrence of each of the categories. Therefore, it is possible for the model to predict multiple NBS measures as potential solutions at the same time. The training process was continued until the validation error converged. The ANN model takes in the output of the transforming function in the previous stage and provides the user with a list of possible NBS measure(s) to implement. The architecture of the ANN model is presented in Fig. 5.

The model was trained and tested on the case repository. This model solves a multi-label classification problem. Each case is associated with multiple NBS measures as class labels, making the accuracy measurement more complicated than single-label classification models [17]. Considering the multi-label structure of the model's prediction, a micro-averaged F1-score (Micro-F1) is used as a label-based metric as well as hamming loss and subset accuracy as two example-based metrics (see Table 2) to assess the model's performance [18]. F1-score is a commonly used metric for comparing the performance of two classifiers [16]. Subset accuracy evaluates the fraction of correctly classified labels (identical to the ground truth), which is usually an overly strict metric for multi-label classification models. The hamming loss evaluates performance based on the instance-label pair prediction errors; error here means a relevant label is missed or an irrelevant label is predicted [18]. The performance of the ANN model is assessed against a Support Vector Machine (SVM) model which is mixed with the TF-IDF vectorizer and trained on the same dataset. The ANN model shows better performance according to all three indicators. For more information regarding the performance of multi-label classification models see Wang et al. [16], Tsoumakas & Katakis [15], and Li et al. [5].

**Table 2:** ANN model vs. SVM model performance.

Performance metric	Micro-F1	Subset accuracy	Hamming loss
ANN	0.7127	0.3227	0.1169
SVM+TF-IDF	0.5712	0.2272	0.1801

### Case retrieval

The first task of the CBS at this stage is to filter cases in the case repository based on the hypothesis generated by the ANN classifier. Users need to select at least one of the NBS measures predicted by the ANN model, and then through filtering, only cases that have implemented the selected NBS measure(s) are considered for further analysis. Subsequently, the system evaluates the similarity between the target case and the filtered cases from the repository. The similarity between every two cases (*S*) is calculated as a function of similarities (*Sim*) between their problem features (*F*):

$$S=\sum_{j=1}^{6}w_{j}Sim\left(F_{ij},F_{tj}\right)$$

In this equation, S is the weighted sum of the similarities between the target case and a case in the case repository, *w_j_* is the weight of the feature, and *Sim(F_ij,_ F_tj_)* is the similarity between the target case (*t*) and the *i_th_* case in the repository at the *j_th_* feature. The system allows users to define a custom weight for each feature *(w_j_)*; otherwise, equal weight is considered for all features. Considering the type of features, four different similarity measurement methods were used (Table 3). For four of the six problem features, the similarity is calculated as a function of the distance between the features:

$$Sim\left(F_{ij},F_{tj}\right)=1-Dist\left(F_{ij},F_{tj}\right)$$

In the latter equation, *Dist(F_ij,_ F_tj_)* is the normalized distance between the target case (*t*) and the *i_th_* case in the repository at the *j_th_* feature. Appendix B provides a more extensive description of each similarity and distance measurement method.

**Table 3:** Similarity measures for problem features.

Feature	Data type	Similarity measure
• Challenges addressed • Project Initiators	Multi-label categorical	(1 - Normalized hamming distance)
• Climate zone	Multi-class categorical	Matching similarity
• Scale • Cost	Ordinal	(1 - Euclidian distance)
• Project description	Textual	Cosine similarity

After calculating the similarity between the target case and historical cases, the system selects and presents the *K* most similar cases to the users. By default, the value of *K* is set to five, but the user is able to customize this value.

### Adaption and retain

Adaption is the process of transforming the recommendations provided by the system into solutions suitable for the problem at hand [6]. This adaption process is a challenging task in many CBS, especially when situated in an urban environment in which many variables and formal as well as informal information sources affect the decision-making process. Similar cases and recommended solutions will not exactly match the target case's requirements and thus need to be tailored and adapted to the target context. Therefore, the NBS-CBS relies on human reasoning to adapt the recommendations to the specific target context. The retaining process in NBS-CBS includes adding the problem solved to the case repository. The UNA handles the retainment.

## Method validation

**Fig. 6:**
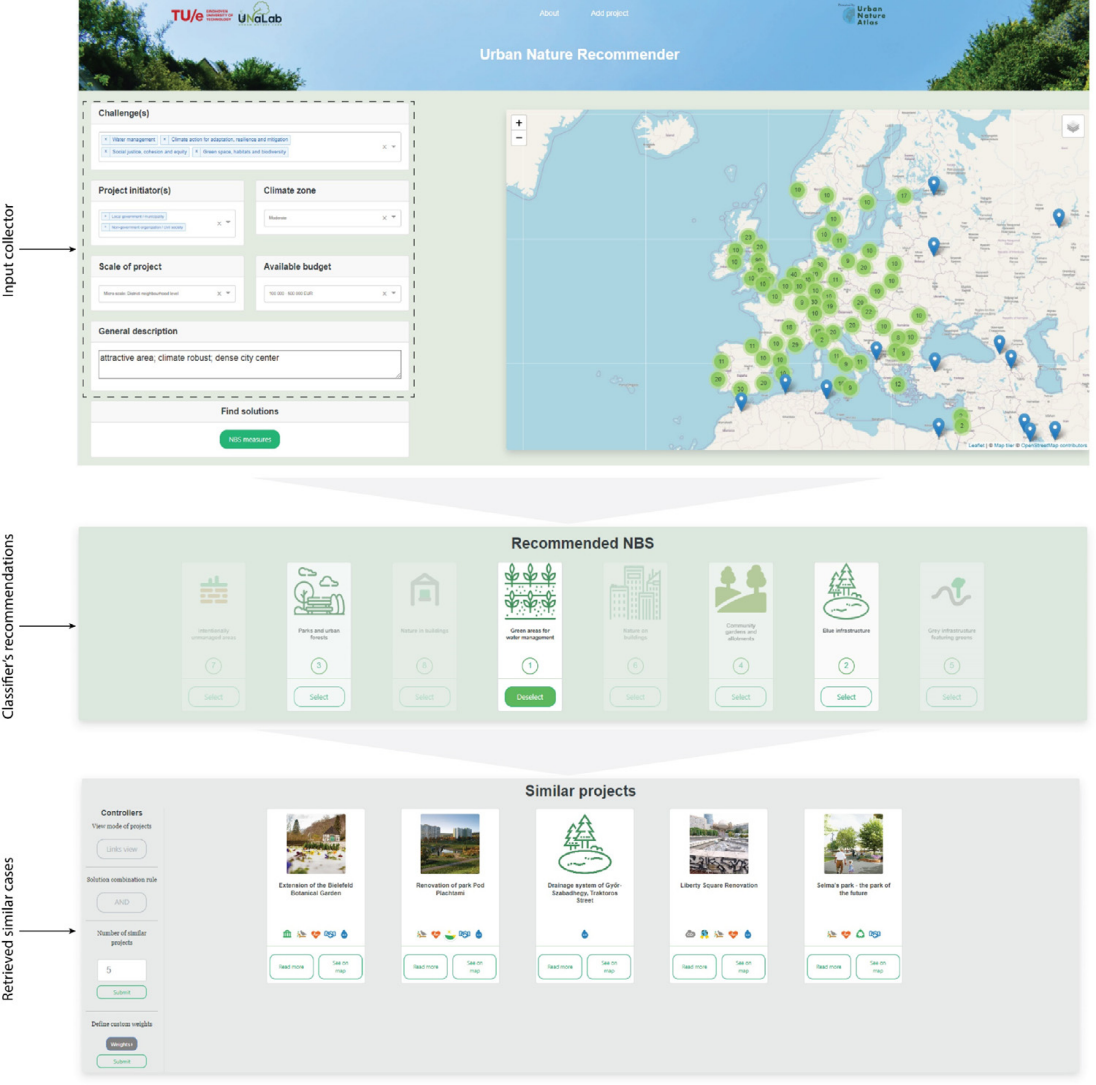
NBS-CBS test results: target case 1.

**Fig. 7:**
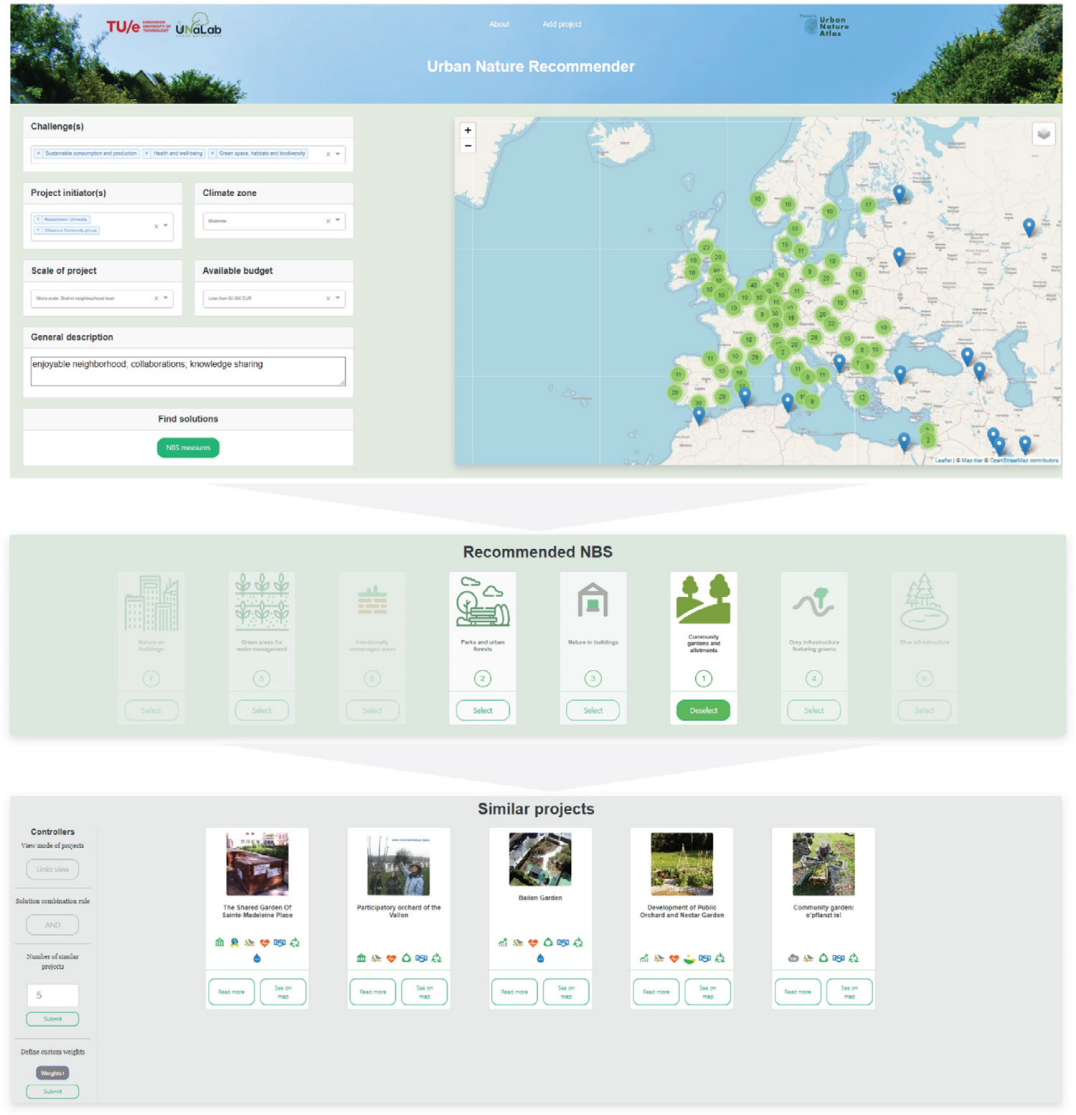
NBS-CBS test results-target case 2.

NBS-CBS was tested with a group of seven experts from the municipality of Eindhoven and the Eindhoven University of Technology to assess the performance and validity of this system in practice. A More detailed explanation of this test session is provided in the related research article [13]. In the test session, experts designed two target cases considering the context of Eindhoven to find solutions and similar cases. The steps taken in this test are presented in Fig. 6 and Fig. 7. The experts found that the NBS-CBS provides a more effective approach for inferring relevant lessons from the repository. They found the NBS recommended to be aligned with their expectations and the identified cases to have similar conditions compared to their target case.

## Conclusion

NBS-CBS facilitates the knowledge acquisition from the UNA NBS project repository using a hybrid architecture, combining a black-box with a white-box model. The step-wise framework of the NBS-CBS guides the process of finding relevant information from an extensive experience repository. The intuitive structure of this system and its evidence-based recommendations make it useful for a wide range of stakeholders, including the ones with a limited background knowledge regarding NBS. The hybrid structure of the system appears to achieve a balance between performance and understandability. The system relies on an ANN model to find a solution and engage users by allowing them to assess the relevance of solution(s) by scrutinizing similar cases. The NBS-CBS can be further improved in the future. For example, more advanced classification models and text vectorization methods that can consider combination of words can be applied to further improve the performance and accuracy of the system.

## CRediT authorship contribution statement

**Shahryar Sarabi:** Conceptualization, Methodology, Formal analysis, Writing – original draft. **Qi Han:** Supervision, Writing – review & editing. **Bauke de Vries:** Supervision, Writing – review & editing. **A.Georges L. Romme:** Supervision, Writing – review & editing.

## Declaration of Competing Interest

The authors declare that they have no known competing financial interests or personal relationships that could have appeared to influence the work reported in this paper.

## Data Availability

The authors do not have permission to share data.

## Appendix Supplementary materials

Image, application 1
