# Supplementary material for: Methodology for development of an expert system to derive knowledge from existing nature-based solutions experiences
Source: MethodsX. 2022 Dec 21;10:101978. doi: 10.1016/j.mex.2022.101978 (PMC9816772; doi:10.1016/j.mex.2022.101978)
Supplement: Supplementary file 1 [file mmc1.docx]

# Appendix A: Text mining

Each case in the case repository includes a general description feature which is a textual feature providing information that could not be recorded by means of tabular features. However, to use the text data for the classification and case similarity assessment, it should go through some transformations. Text mining provides the opportunity to analyze and process unstructured text data (Miner, 2012). In this study, text mining is used to retrieve the information from cases and transform the text into structured data to be used by the Neural network model and assist in case retrieval.

The vector-space model is the most popular method to represent text in a structured format that can be processed by computers. In this model, a text is represented by a matrix with each cell indicating the frequency of a word in the text; before vectorizing this text, it needs to go through a few preprocessing steps.

## Preprocessing

### Convert to lower case

At first, all the words in a text need to be converted into lower case font, since upper and lowercase texts are treated differently by the machine. For example, green and Green are treated differently, so one needs to transform all text to lowercase.

### Removing stop words, punctuation, and single characters

Stop words are the words occurring most commonly, which do not add any new information and removing them will increase the computation and storage efficiency. NLTK library in python provides a direct method for retrieving the stop words.

Punctuations are unnecessary symbols in text documents, and they need to be removed.

Single characters do not add useful information; therefore, all words with a length not greater than one character are removed.

### Convert numbers to text

Machine treats a number's numerical and textual representation differently, while they mean the same. Therefore, converting all numbers in the corpus to words is needed. For example, 120 is transformed into "one hundred and twenty".

### Stemming

Stemming is the process of converting related words to a single standard format or their stem. For example, "walking" and "walked" both refer to the same action. Stemming includes the identification and removal of prefixes and suffixes. Stemming increases the accuracy of text mining by reducing the number of distinct types (Shen et al., 2017). In this study, we use Porter stemmer, the most popular stemming algorithm.

## Vectorizing

In this step, the text for all cases is transformed into a matrix where each column represents a unique word. Therefore, all case texts share the same set of representative textual features (Shen et al., 2017). Then a value should be assigned to each cell of the matrix representing the relative importance of a word in a case text. For this purpose, we are using the term frequency-inverse document frequency (TF-IDF) approach to measure and represent the importance of each word in the document collection (Manning & Schutze, 1999).

Words occurring in a high frequency can possibly be used to represent the content of a document. However, there are words in the entire document collection, like 'green', which not only occur several times in a single case text but also in many other texts. Therefore, a term like 'green' cannot help distinguish between the solutions or find similar cases. Thus, the word frequency in the whole document library is used to offset the term frequency. The TF-IDF value of each term can be calculated as follows:

| *TF_IDF_ij_*=  (1+*log(TF_ij_^­^))log(N/DF_j_*) *if TF_ij_* ≥ 1  0 *if TF_ij_* = 0 | Eq. (A.1) |
| --- | --- |

Where TF_IDF denotes the value of the term *j* in the case text *i*; *TF_ij_^­^*  denotes the frequency of the term *j* in the case text *i; DF_j_* Denotes the number of documents containing the term *j* in the document library. *N* is the total number of documents (cases).

The TF-IDF vectorizer from the scikit-learn python library (Pedregosa et al., 2011) is used to perform the vectorization task. The TF-IDF vectorizer is trained on the whole document collection and saved to be reused. The number of words in vocabulary is set to 6000 by the author. When a new text is fed into the trained vectorizer, it provides an array of TF-IDF values with a length of 6000. Each element of this array refers to a unique word.

# Appendix B- distance and similarity measurement methods

## Normalized Hamming distance

Hamming distance is a measure used to compare strings of equal length. The hamming distance is the number of positions where the corresponding symbols are different (Hamming, 1950). Hamming distance is a commonly used method to measure the distance between categorical variables. For example, the hamming distance between the challenge feature of the target case with a historical case is:

Dist(100010100010, 110000000011)=4

The normalized Hamming distance divided the hamming distance by the number of bits, so for the example mentioned above, it will be 4/12=0.333.

## Matching similarity

In this method, the similarity between two values is 1 when they are the same and 0 when they are different. This method measures similarity in features with a multi-class categorical variable, such as “climate zone.” Thus:

| *Sim(F_ij,_ F_tj_)=*  1  0  *If F_ij_ = F_tj_*  *If F_ij_ ≠ F_tj_* | Eq. (B.1) |
| --- | --- |

where *Sim(F_ij,_ F_tj_)* is the similarity between *ith* case from the library and the target case (*t)* with regards to the feature *j*.

## Euclidian distance

The Euclidean distance is used to measure the similarity between features with crisp numerical values (cost and scale). The shorter the distance, the more similar the case from the case-repository is to the target case. Euclidean distance is measured as follows:

| $Dist\left( F_{ij},F_{tj} \right)= \sqrt{{{(F}_{ij}-F_{tj})}^{2}}$ | Eq. (B.2) |
| --- | --- |

Where *Dist(F_ij,_ F_tj_)* is the distance between *i*th case from the library and the target case (*t)* with regards to the feature *j*.

## Cosine similarity

Cosine similarity is commonly used to measure the similarity between two (document) vectors by finding the angle between the two vectors. The cosine distance between two text vectors is calculated as follows:

| $Sim\left( F_{i},F_{t} \right)= \frac{\sum_{j=1}^{n} W_{ij}W_{tj}}{\sqrt{\sum_{j=1}^{n} {W_{ij}}^{2} \sum_{j=1}^{n} {W_{tj}}^{2}}}$ | Eq. (B.3) |
| --- | --- |

Where *Sim(F_i,_ F_t_)* denotes the cosine similarity between the textual features of the *i*th case from the case library(*F_i_*) and the target case (*F_t_*), *W_ij_* denotes the TF-IDF value of the *j*th term of the text vector of the *i*th case from the case library, and *W_tj_* denotes the TF-IDF value of the *j*th term of the text vector of the target case.

# References

Hamming, R. W. (1950). Error detecting and error correcting codes. *The Bell System Technical Journal*, *29*(2), 147–160. https://doi.org/10.1002/j.1538-7305.1950.tb00463.x

Manning, C., & Schutze, H. (1999). *Foundations of statistical natural language processing*. MIT press.

Miner, G. (2012). *Practical text mining and statistical analysis for non-structured text data applications*. Academic Press.

Pedregosa, F., Varoquaux, G., Gramfort, A., Michel, V., Thirion, B., Grisel, O., Blondel, M., Prettenhofer, P., Weiss, R., Dubourg, V., Vanderplas, J., Passos, A., Cournapeau, D., Brucher, M., Perrot, M., & Duchesnay, É. (2011). Scikit-learn: Machine Learning in Python. *Journal of Machine Learning Research*, *12*(85), 2825–2830. http://jmlr.org/papers/v12/pedregosa11a.html

Shen, L., Yan, H., Fan, H., Wu, Y., & Zhang, Y. (2017). An integrated system of text mining technique and case-based reasoning (TM-CBR) for supporting green building design. *Building and Environment*, *124*, 388–401. https://doi.org/https://doi.org/10.1016/j.buildenv.2017.08.026
